# Prolactinoma-associated headache: Clinical and radiological correlates and long-term outcomes

**DOI:** 10.1007/s11102-026-01699-1

**Published:** 2026-05-23

**Authors:** Lukas Andereggen, Christoph Schankin, Philipp Schuetz, Andrea Stieger, Markus M. Luedi, Atila Cihan, Michel Roethlisberger, Luigi Mariani, Jürgen Beck, Emanuel Christ

**Affiliations:** 1 Department of Neurosurgery, Kantonsspital Aarau, Aarau, Switzerland; 2 Faculty of Medicine, University of Bern, Bern, Switzerland; 3 Department of Neurology, Inselspital, Bern University Hospital, University of Bern, Bern, Switzerland; 4 Centre for Migraine and Headache, Bellevue Medical Group, Zurich, Switzerland; 5 Medical University Department, Clinic for Endocrinology, Diabetes and Metabolism, Kantonsspital Aarau, Aarau, Switzerland; 6 Medical Faculty of the University of Basel, Basel, Switzerland; 7 Department of Anaesthesiology and Pain Medicine, Kantonsspital St. Gallen, St. Gallen, Switzerland; 8 Department of Endocrinology, Diabetes and Metabolism, University Hospital of Basel, Basel, Switzerland; 9 Department of Neurosurgery, University Hospital of Basel, Basel, Switzerland; 10 Department of Neurosurgery, Medical Center, University of Freiburg, Freiburg, Germany

**Keywords:** Prolactinoma, Headache, Cavernous sinus invasion, Dopamine agonist, Transsphenoidal surgery

## Abstract

**Purpose:**

While headache is frequently encountered in patients with prolactinomas, data on its determinants and long-term outcome following upfront medical versus upfront surgical treatment are limited. We aimed to identify clinical and radiological determinants of prolactinoma-associated headache (PAH) and to evaluate long-term headache outcomes according to the primary treatment strategy.

**Methods:**

We retrospectively analyzed 145 prolactinoma patients treated at a tertiary center. Headache at presentation, endocrine parameters, tumor size, and cavernous sinus involvement were assessed. Independent determinants of PAH at diagnosis were identified using multivariable logistic regression. Long-term headache status and prolactin outcomes were evaluated according to upfront dopamine agonist (DA) therapy or transsphenoidal surgery (TSS).

**Results:**

At diagnosis, 42 of 145 patients (28.9%) presented with PAH. Compared with patients without PAH, those with PAH were older, more often male, and more likely to harbor macroadenomas and tumors with cavernous sinus involvement. In multivariable analysis, cavernous sinus involvement was the only independent factor associated with PAH (adjusted OR, 7.8; 95% CI, 1.2–48.7; *p* = 0.03), whereas baseline prolactin levels were not independently associated. After a median follow-up of 80 months, headache prevalence declined from 28.9% to 2.8% (*p* < 0.001). Durable headache improvement was observed after both DA therapy and transsphenoidal surgery, with no significant difference between treatment modalities.

**Conclusion:**

PAH is most strongly linked to tumor invasiveness rather than to prolactin levels alone. Durable headache relief is achieved with both upfront surgical and medical therapy, suggesting that effective tumor control, rather than the specific initial treatment approach, is the key determinant of headache improvement. These findings support careful patient counseling and underscore the importance of an individualized multidisciplinary approach in prolactinoma patients presenting with headache.

## Introduction

Headache associated with pituitary tumors is a well-recognized clinical phenomenon, yet its underlying mechanisms are not fully understood [1, 2]. Both structural and biochemical factors have been proposed in pituitary adenoma-related headaches. From a structural perspective, large adenomas may cause pain by stretching the dura within the sella turcica, while lateral extension into the cavernous sinus may involve pain-sensitive structures such as the trigeminal nerve or the cavernous carotid artery [3, 4]. In patients with non-functioning pituitary adenomas, cavernous sinus invasion has been identified as an important risk factor for headache [5]. In hormone-secreting pituitary adenomas, headache is likewise frequently encountered [6], yet the specific pathophysiology of prolactinoma-associated headache (PAH) remains poorly characterized [7–9].

This is also consistent with observations in other functioning pituitary adenomas, such as acromegaly, where headache may be clinically prominent despite an incompletely understood pathophysiology, suggesting that both structural and endocrine mechanisms may contribute [10].

Previous reports have suggested that hyperprolactinemia or other neuroendocrine mechanisms may contribute to headache in prolactinoma patients [7, 8, 11]. This hypothesis is supported by observations that dopamine agonist (DA) therapy often leads to headache improvement [7]. In addition, several case reports describe resolution of severe headache syndromes, including cluster headache–like or short-lasting unilateral neuralgiform headache attacks with conjunctival injection and tearing (SUNCT) presentations, following DA treatment [8, 12–14]. Conversely, surgical decompression by transsphenoidal surgery (TSS) has also been shown to significantly alleviate headache in patients with pituitary adenomas [15–18]. Despite these observations, it remains unclear whether headache relief in prolactinoma patients is primarily related to endocrine control, structural tumor decompression, or a combination of both. Importantly, data directly comparing headache characteristics and long-term outcomes following upfront medical versus upfront surgical treatment are scarce. Accordingly, available data to support counseling of prolactinoma patients presenting with significant headache symptoms are sparse, particularly with respect to long-term outcomes under different upfront treatment strategies. Against this background, we investigated PAH in a large, homogeneous, single-center cohort. Specifically, we aimed to (i) characterize the clinical profile of prolactinoma patients presenting with headache, (ii) identify clinical and radiological factors associated with PAH, and (iii) evaluate long-term headache outcomes following primary medical or primary surgical treatment, thereby clarifying the relative contribution of structural versus endocrine factors.

## Methods

### Study design and patient selection

We conducted a retrospective observational study of patients with prolactinomas treated at a tertiary referral center between 1997 and 2015. Clinical, endocrine, and imaging data were obtained from a prospectively maintained pituitary database and supplemented by detailed medical record review. Patients were managed with either upfront DA therapy or upfront transsphenoidal surgery as the initial treatment strategy; treatment allocation reflected tumor characteristics, interdisciplinary decision-making, and patient preference.

Exclusion criteria were age < 18 years at diagnosis, follow-up < 12 months, mixed-secreting pituitary adenomas, and secondary hyperprolactinemia (e.g., medication-induced or other non-adenomatous causes). The diagnosis of prolactinoma was established based on an integrated clinical, biochemical, and radiological assessment performed at a dedicated pituitary center. This included characteristic clinical features, persistently elevated serum prolactin levels, and radiological evidence of a pituitary adenoma on MRI. Macroprolactinemia was assessed where clinically indicated (i.e. high prolactin levels without any impact on gonadotrophic axis). To ensure accurate classification, cases considered more consistent with a non-functioning pituitary adenoma with stalk effect were not included. In particular, patients with prolactin levels judged disproportionate to tumor size or with imaging findings suggestive of stalk compression rather than a prolactin-secreting lesion were not classified as having a prolactinoma. No formal sample size calculation was performed; instead, all eligible patients meeting the inclusion criteria during the study period were included. Because of the long retrospective study period, complete screening data for all patients assessed for eligibility were not consistently available. However, all patients fulfilling the predefined inclusion and exclusion criteria were included in the final analysis. Headache was identified in the medical records at presentation and during follow-up.^10^ To minimize confounding, patients were excluded if headache symptoms were considered primarily attributable to an alternative diagnosis, including secondary intracranial causes (e.g., recent traumatic brain injury, intracranial hemorrhage, CNS infection, or giant cell arteritis) or a documented primary headache disorder (e.g., migraine or trigeminal neuralgia) unrelated to the pituitary lesion. Patients with medical comorbidities precluding one treatment modality were managed individually and were not excluded if all other criteria were met. The study was approved by the Human Research Ethics Committee of Bern (KEK Bern; reference numbers KEK 10-10-2006 and KEK 8-11-2006) and conducted in accordance with the Declaration of Helsinki; the requirement for informed consent was waived due to the retrospective design.

### Clinical and endocrine assessments

Baseline patient characteristics included age, sex, body mass index (BMI), presenting symptoms, and relevant medical history, including a family history of chronic headache disorders. Headache at presentation was classified as PAH when a recurrent or persistent headache constituted a chief complaint or a major symptom leading to diagnostic evaluation and was not attributable to an alternative cause. This definition was chosen to capture clinically meaningful headache burden rather than incidental or nonspecific symptoms.

Baseline pituitary function was assessed in all patients. Hypogonadotropic hypogonadism was defined by reduced sex steroid concentrations (testosterone in men, estradiol in women) in the presence of inappropriately low or normal gonadotropin (LH/FSH) levels. Secondary adrenal insufficiency and central hypothyroidism were defined by low serum cortisol or free thyroxine (fT4) levels with low or inappropriately normal ACTH or TSH concentrations, respectively.

Serum prolactin levels were measured using standard immunoassays; samples with very high prolactin concentrations were reassessed after serial dilution to exclude a hook effect [19, 20]. The upper limit of normal prolactin was defined as 20 µg/L for both sexes [21]. Clinical manifestations of hyperprolactinemia, including galactorrhea, menstrual disturbances, and sexual dysfunction, were systematically recorded.

### Radiological assessment

All patients underwent pituitary magnetic resonance imaging (MRI) at diagnosis using 1.5T or 3T scanners with dedicated sellar protocols, including dynamic contrast-enhanced sequences [22]. Radiological assessments were based on routine clinical imaging reports obtained at diagnosis and were performed independently of the present study question, without systematic knowledge of headache status. Tumor size was determined on baseline MRI and classified as microadenoma (< 10 mm maximal diameter) or macroadenoma (≥ 10 mm). Adenoma invasiveness was assessed using the Knosp grading system to evaluate cavernous sinus involvement. For analytical purposes, invasiveness was dichotomized and defined as Knosp grade ≥ 1, reflecting any radiological contact or extension toward the cavernous sinus. Visual field examination was performed in patients with suprasellar tumor extension to assess for optic chiasm compression.

### Treatment approach

The choice of primary treatment strategy with upfront DA therapy or upfront transsphenoidal surgery (TSS) was determined by an interdisciplinary pituitary tumor board, taking into account tumor characteristics (i.e., cavernous sinus infiltration), endocrine status, clinical presentation, and patient preference, including the desire to avoid long-term dependence on DAs [16], [23, 24], [25]. Treatment allocation, therefore, reflected real-world clinical decision-making rather than random assignment. First-line medical therapy consisted of dopamine agonists, including bromocriptine, quinagolide, and cabergoline, with the specific agent selected according to clinical practice and availability during the study period. First-line surgical treatment comprised endonasal TSS performed by experienced pituitary neurosurgeons, using either a microscopic or endoscopic technique. In cases of intraoperative cerebrospinal fluid leakage, sellar reconstruction was performed using a multilayer closure technique (e.g., synthetic graft, gelatin sponge, and fibrin sealant) [26, 27].

Post-treatment follow-up included regular clinical assessments, endocrine evaluation (including prolactin levels), and serial pituitary MRI. Headache outcome was assessed retrospectively from follow-up documentation and categorized as resolved, improved, unchanged, or worsened according to patient report. For statistical analysis, the presence of persistent headache at the last follow-up was used as the primary headache outcome measure; headache remission was defined as the absence of clinically relevant headache symptoms at the final follow-up. For the purpose of analysis, early follow-up was defined as the first post-treatment clinical and endocrine assessment after initiation of therapy (median 5 months [IQR 3–8]). Last follow-up was defined as the last documented clinical and endocrine assessment available for each patient.

### Statistical analysis

Analyses were performed on a complete-case basis; missing data were not imputed. Continuous variables were assessed for normality using the Shapiro–Wilk test and are reported as mean ± standard deviation (SD) for approximately normally distributed data or as median with interquartile range (IQR) for skewed distributions. Categorical variables are presented as counts and percentages. Between-group comparisons were performed using Student’s *t* test or the Mann–Whitney *U* test, as appropriate. Within-group changes over time (e.g., baseline vs. last follow-up prolactin) were analyzed using the Wilcoxon signed-rank test. Categorical variables were compared using Pearson’s chi-square test or Fisher’s exact test, depending on expected cell counts.

To identify factors associated with PAH at diagnosis, univariable logistic regression analyses were performed with PAH as the dependent (outcome) variable. Candidate predictors were selected a priori based on clinical plausibility and included age, sex, family history of chronic headache disorders, baseline BMI, baseline prolactin level, tumor size category (macroadenoma vs. microadenoma), and cavernous sinus involvement. Cavernous sinus involvement was assessed using the Knosp grading system and dichotomized for analysis as absent (Knosp 0) or present (Knosp ≥ 1). Because baseline prolactin concentrations were markedly right-skewed, prolactin values were log-transformed prior to regression modeling. Variables with *p* ≤ 0.05 in univariable analysis were entered into a multivariable logistic regression model to estimate independent associations with PAH. Multicollinearity among variables entered into the multivariable model was assessed using collinearity diagnostics and was not considered to materially affect model stability or interpretability. Results are reported as odds ratios (ORs) with 95% confidence intervals (CIs). All tests were two-sided, and *p* < 0.05 was considered statistically significant. No adjustment for multiple testing was performed.

## Results

### Baseline clinical and tumor characteristics

A total of 145 patients with prolactin-secreting pituitary adenomas met the inclusion criteria. At diagnosis, 42 patients (28.9%) presented with PAH, whereas 103 patients (71.1%) did not report clinically relevant headache. Baseline characteristics stratified by headache status are summarized in Table 1. Patients with PAH were significantly older at diagnosis than those without PAH (43.7 ± 15.3 vs. 35.9 ± 13.4 years; *p* = 0.003). The proportion of women was lower in the PAH group than in the non-PAH group (57.1% vs. 75.7%; *p* = 0.04). Notably, PAH remained common among women, affecting 24 of 102 female patients (23.5%). Primary treatment allocation is summarized in Table 2. Overall, 64 patients received upfront dopamine agonist therapy (Table 3) and 81 underwent upfront transsphenoidal surgery. Patients with cavernous sinus involvement were more frequently treated with upfront dopamine agonist therapy than those without cavernous sinus involvement (32/42, 76.2% vs. 28/87, 32.2%; *p* < 0.001). Upfront dopamine agonist therapy was also more common in men than in women (29/43, 67.4% vs. 35/102, 34.3%; *p* < 0.001), whereas treatment allocation did not differ significantly by tumor size category (*p* = 0.20).

**Table 1:** Clinical and tumor characteristics of prolactinoma patients with and without prolactinoma-associated headache (PAH) at diagnosis

Characteristic	Without PAH (*n* = 103)	With PAH (*n* = 42)	All patients (*n* = 145)	*p*-value
Demographics				
Age at diagnosis, years (mean ± SD)	35.9 ± 13.4	43.7 ± 15.3	38.1 ± 14.3	0.003
Female sex, *n* (%)	78 (75.7%)	24 (57.1%)	102 (70.3%)	0.04
Body mass index, kg/m² (mean ± SD)	26.3 ± 5.8	28.2 ± 4.6	26.9 ± 5.5	0.11
Tumor characteristics				
Macroprolactinoma (≥ 10 mm), *n/N *(%)	44 /103 (42.7%)	30 / 42 (71.4%)	74 / 145 (51.0%)	0.002
Cavernous sinus involvement, *n/N* (%)	20 / 91 (22.0%)	22 / 38 (57.9%)	42 / 129 (32.6%)	< 0.001
Visual field deficits, *n/N* (%)	2 / 103 (1.9%)	0 / 42 (0.0%)	2 / 145 (1.4%)	1.00
Baseline prolactin, µg/L (median [IQR])	196.9 [89.7–779.8]	686.0 [167.0–2941.0]	218.2 [99.9–1184.3]	0.02
Endocrine dysfunction				
Hypogonadotropic hypogonadism, *n/N* (%)	69 / 77 (89.6%)	19 / 21(90.5%)	88 / 98(89.8%)	0.99
Central hypothyroidism, *n/N* (%)	6 / 92 (6.5%)	4 / 39 (10.3%)	10 / 131 (7.6%)	0.48
Somatotroph deficiency, *n/N* (%)	5 / 86 (5.8%)	1 / 31 (3.2%)	6 / 117 (5.1%)	1.00
Secondary adrenal insufficiency, *n/N* (%)	4 / 92 (4.3%)	5 / 39 (12.8%)	9 / 131 (6.9%)	0.13
Clinical presentation				
Galactorrhea, *n/N* (%)	37 / 75 (49.3%)	12 / 23 (52.2%)	49 / 98 (50.0%)	1.00
Amenorrhea / menstrual disturbance, *n/N* (%)	55 / 70 (78.6%)	12 / 17 (70.6%)	67 / 87 (77.0%)	0.70
Infertility, *n/N* (%)	21 / 37 (56.8%)	5 / 8 (62.5%)	26 / 45 (57.8%)	1.00
Low libido,* n/N* (%)	16 / 24 (66.7%)	14 / 17 (82.4%)	30 / 41 (73.2%)	0.31
Erectile dysfunction,* n/N* (%)	11 / 23 (47.8%)	11 / 16 (68.8%)	22 / 39 (56.4%)	0.33

Values are presented as mean ± SD, median [interquartile range], or number (%), as appropriate

**Table 2:** Primary treatment strategy according to sex, tumor size, and cavernous sinus involvement

A. Characteristic	Upfront DA *n*/*N* (%)	Upfront TSS *n*/*N* (%)	*p*-value
Overall cohort	64/145 (44.1%)	81/145 (55.9%)	–
Sex			
Female	35/102 (34.3%)	67/102 (65.7%)	
Male	29/43 (67.4%)	14/43 (32.6%)	< 0.001
Tumor size			
Microprolactinoma	27/71 (38.0%)	44/71 (62.0%)	
Macroprolactinoma	37/74 (50.0%)	37/74 (50.0%)	0.20
Cavernous sinus involvement			
Absent	28/87 (32.2%)	59/87 (67.8%)	
Present	32/42 (76.2%)	10/42 (23.8%)	< 0.001

*DA* dopamine agonist, *TSS* transsphenoidal surgery

**Table 3:** Baseline dopamine agonist subtype and dose among patients receiving upfront dopamine agonist therapy

Dopamine agonist	*n*/*N* (%)	Interpretable baseline dose, *n*/*N*	Dose summary
Bromocriptine (Parlodel)	27/64 (42.2%)	15/27	Median 7.5 mg/day
Quinagolide (Norprolac)	17/64 (26.6%)	16/17	Median 75 µg/day
Cabergoline (Dostinex/Cabaser)	13/64 (20.3%)	7/13	Median 1.5 mg/week
Subtype not unequivocally retrievable	7/64 (10.9%)	–	–

Tumor characteristics also differed between groups. Macroprolactinomas were more frequent in patients with PAH (30/42, 71.4%) than in those without PAH (44/103, 42.7%; *p* = 0.002). Among the 129 of 145 patients (89%) with available baseline MRI data allowing assessment of cavernous sinus involvement, radiological cavernous sinus involvement was significantly more frequent in patients with PAH than in those without PAH (22/38, 57.9% vs. 20/91, 22.0%; *p* < 0.001). Baseline prolactin levels were also higher in the PAH group (median 686.0 µg/L [IQR 167.0–2941.0]) than in the non-PAH group (median 196.9 µg/L [IQR 89.7–779.8]; *p* = 0.02), although with substantial overlap between groups. Hypogonadotropic hypogonadism was highly prevalent and comparable in both groups (PAH 91% vs. non-PAH 90%; *p* = 0.99). In contrast, BMI did not differ significantly between patients with and without PAH (28.2 ± 4.6 vs. 26.3 ± 5.8 kg/m²; *p* = 0.11), and visual field deficits were rare overall (2/145, 1.4%). Other pituitary deficiencies, including central hypothyroidism, somatotroph deficiency, and secondary adrenal insufficiency, did not differ significantly between groups. Likewise, clinical manifestations of hyperprolactinemia, including galactorrhea, amenorrhea or menstrual disturbance, infertility, low libido, and erectile dysfunction, were broadly similar in patients with and without PAH (Table 1).

### Factors associated with PAH at diagnosis

We next evaluated determinants of PAH at diagnosis. In univariable logistic regression, older age, male sex, macroadenoma status, cavernous sinus involvement, and baseline PRL levels were each associated with PAH. In the multivariable model, cavernous sinus involvement remained the only independent predictor of PAH (adjusted OR, 7.8; 95% CI, 1.2–48.7; *p* = 0.03), whereas tumor size category and baseline prolactin levels did not retain independent associations after adjustment (Table 4). These findings indicate that radiological invasiveness represents the strongest independent correlate of PAH at presentation.

**Table 4:** Univariable and multivariable logistic regression analysis for prolactinoma-associated headache at diagnosis

Variable	Univariable OR (95% CI)	*p*-value	Multivariable OR (95% CI)	*p*-value
Age	1.04 (1.01–1.07)	0.003	1.02 (0.98–1.06)	0.31
Male sex	2.3 (1.1–4.8)	0.03	2.1 (0.5–8.4)	0.30
Macroadenoma (≥ 10 mm)	3.4 (1.6–7.3)	0.002	2.3 (0.7–7.4)	0.16
Cavernous sinus invasion	4.9 (2.2–10.9)	< 0.001	7.8 (1.2–48.7)	0.03
Baseline prolactin (log-transformed)	2.2 (1.3–3.7)	0.003	1.0 (0.7–1.4)	0.92
Body mass index (BMI)	1.05 (0.98–1.12)	0.15	–	–
Family history of headache	1.1 (0.4–3.1)	0.86	–	–

Odds ratios (ORs) are shown with 95% confidence intervals (CI). Variables with *p* ≤ 0.05 in univariable analysis were entered into the multivariable model

### Long-term endocrine and headache outcomes

**Fig. 1:**
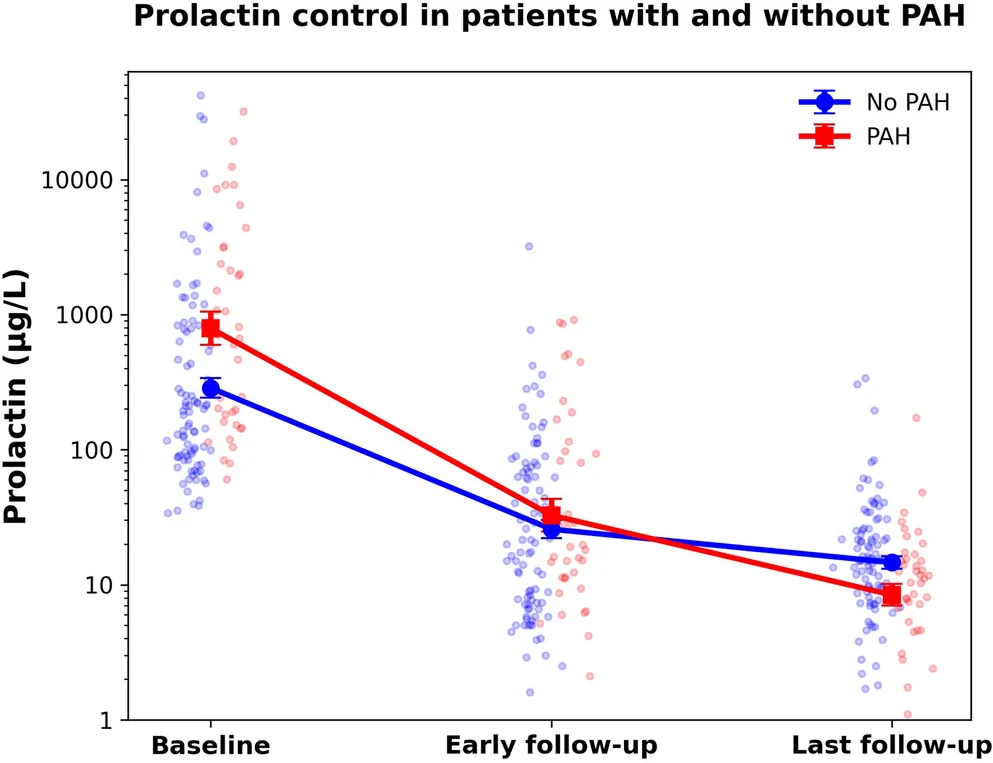
Prolactin control in patients with and without PAH

Median follow-up was 80 months (IQR 39–152 months). Treatment resulted in effective long-term control of hyperprolactinemia in the majority of patients. Median serum prolactin levels declined from 218 µg/L at diagnosis to 14 µg/L at last follow-up (*p* < 0.001), and prolactin normalization was achieved in 118 of 145 patients (85%). Rates of prolactin normalization did not differ significantly between patients presenting with and without PAH (93% vs. 81%, *p* = 0.12, Fig. 1), nor between patients treated with upfront TSS and those treated with upfront DA therapy (90% vs. 79%, *p* = 0.10).

Longitudinal serum prolactin levels at baseline, early follow-up, and last follow-up in prolactinoma patients with prolactinoma-associated headache (PAH) and without PAH. Data are shown as mean ± SEM on a logarithmic scale, with individual patient values overlaid. Prolactin levels declined significantly over time in both groups (*p* < 0.001). At last follow-up, prolactin levels did not differ significantly between patients with and without PAH (*p* = 0.12).

Hypogonadotropic hypogonadism was highly prevalent at diagnosis and comparable between groups (PAH 90.5% vs. non-PAH 89.6%; *p* = 0.99). During follow-up, its prevalence declined substantially in both groups, decreasing to 64.0% in PAH patients and 61.1% in non-PAH patients at early follow-up, and to 26.1% and 30.4%, respectively, at long-term follow-up.

**Fig. 2:**
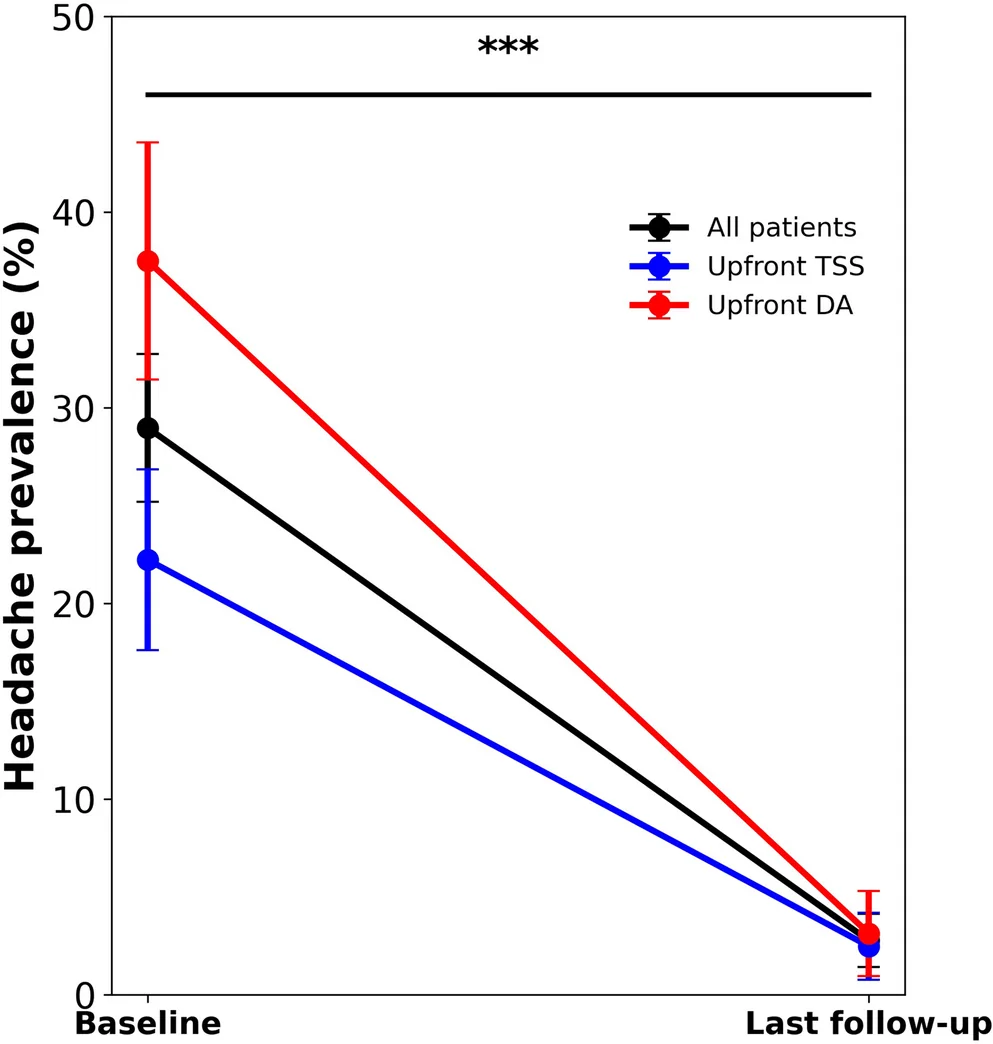
Headache prevalence over time according to primary treatment strategy in prolactinoma patients

Across the entire cohort, the prevalence of headache declined from 28.9% at diagnosis (42/145) to 2.8% at last follow-up (4/145; *p* < 0.001), indicating durable symptom improvement in most patients. Headache outcomes were favorable under both upfront treatment strategies (Fig. 2). Among patients treated with upfront TSS, headache prevalence decreased from 22.2% (18/81) at presentation to 2.5% (2/81) at last follow-up (*p* < 0.001). Similarly, among patients treated with upfront DA therapy, headache prevalence declined from 37.5% (24/64) to 3.1% (2/64; *p* < 0.001). The proportion of patients with persistent headache at the last follow-up did not differ significantly between treatment modalities.

Prevalence of prolactinoma-associated headache at diagnosis (Baseline) and at last follow-up in the overall cohort (black), in patients treated with upfront transsphenoidal surgery (TSS, blue), and in patients treated with upfront dopamine agonist therapy (DA, red). Data are presented as percentages with error bars indicating the standard error of the proportion. Headache prevalence declined significantly over time across all treatment strategies (***p < 0.001).

## Discussion

The present study provides a comprehensive analysis of PAH and its relationship to tumor characteristics and treatment strategy in a large, homogeneous cohort with long-term follow-up. By directly comparing long-term headache outcomes following upfront medical versus upfront surgical treatment, our data extend the existing literature [7, 28, 29] in several important ways. Specifically, we show that (i) PAH is a frequent and clinically relevant presenting symptom, (ii) PAH is most strongly associated with radiological cavernous sinus involvement, rather than with prolactin levels alone, and (iii) long-term headache outcomes are highly favorable after effective prolactinoma treatment irrespective of the initial therapeutic modality. Taken together, these findings support the concept that structural tumor characteristics are clinically more relevant than prolactin excess alone in explaining headache burden at presentation.

In our cohort, clinically significant headache was present in nearly one-third of patients at diagnosis. This is consistent with prior reports indicating that headache is common in pituitary adenomas, including prolactinomas, and may represent a substantial part of the initial symptom burden [29, 30]. Importantly, in our cohort, PAH was not defined as any nonspecific headache mentioned in the chart, but as a clinically meaningful complaint documented as recurrent or persistent and relevant to presentation. This approach was chosen to reflect real-world headache burden in routine prolactinoma care rather than incidental symptoms of uncertain significance. At the same time, because headache was not prospectively assessed as a primary study outcome, the prevalence of PAH at diagnosis may still have been underestimated [8, 9, 31–33]. Our long-term follow-up shows that adequate treatment of the prolactinoma results in substantial improvement or complete resolution of PAH in most patients.

A central finding of this study is the strong association between PAH and cavernous sinus involvement. While macroprolactinomas were more common in patients with PAH, tumor size did not remain independently associated with headache once cavernous sinus involvement was taken into account. This suggests that the anatomical growth pattern of the tumor may be more relevant than size alone [2]. Lateral extension toward the cavernous sinus may increase the likelihood of irritation of pain-sensitive dural and neurovascular structures, including trigeminally innervated tissues and the cavernous carotid region, thereby providing a plausible structural explanation for headache generation. In addition, predominantly intrasellar or suprasellar macroadenomas may cause visual impairment without necessarily provoking headache, unless associated with increased intrasellar pressure or irritation of pain-sensitive structures [3, 4, 34]. Our findings therefore support prior work suggesting that headache in pituitary tumors is more closely related to specific anatomical extension and dural irritation than to tumor bulk alone [3, 29, 35–39]. At the same time, this association should not be interpreted as proof of direct causality. Cavernous sinus involvement may also act as a composite marker of a more invasive tumor phenotype, which could reflect several structural mechanisms relevant to headache. In this context, clinically relevant headache was not restricted to macroprolactinomas, as microprolactinomas with headache were also observed in our cohort. However, macroadenomas and cavernous sinus involvement were substantially more frequent in patients with PAH. Although descriptive, this pattern supports the concept that structurally more invasive tumor phenotypes are more strongly linked to clinically relevant headache. Conversely, because no formal sample size calculation was performed and the number of patients with persistent headache at long-term follow-up was very low, the absence of an independent effect of tumor size should not be overinterpreted as evidence that size has no role at all. Rather, our data suggest that invasiveness provides the stronger discriminatory signal within this cohort.

The lack of an independent association between baseline prolactin levels and PAH in the multivariable model further supports a predominantly structural rather than purely biochemical contribution to headache in prolactinoma patients [28, 40, 41]. Although prolactin and related neuroendocrine pathways have been implicated in pain modulation [42, 43], particularly in migraine and other primary headache disorders, our findings indicate that prolactin excess alone does not adequately explain the occurrence of clinically relevant PAH at the population level. This is an important distinction, because the frequent clinical observation that DA therapy leads to headache improvement is sometimes interpreted as evidence for a direct endocrine mechanism [7, 44]. A similarly mixed structural and endocrine contribution has also been discussed in other functioning pituitary adenomas, such as acromegaly, in which headache may be clinically prominent despite an incompletely understood pathophysiology. In that setting, headache improvement under medical therapy does not always appear to parallel radiological tumor shrinkage, suggesting that biochemical disease activity may also contribute to symptom generation [29, 45]. Our data support a more integrated interpretation: DAs may improve headache not only through prolactin lowering, but also through tumor shrinkage and reduced irritation of pain-sensitive structures [35, 46]. This point also helps to contextualize the overlap between prolactin-related mechanisms and primary headache disorders. Previous studies have suggested a potential association between prolactin and migraine biology, and it is plausible that endocrine factors may modulate headache susceptibility in at least some patients. In the present study, patients with clearly documented primary headache syndromes unrelated to the pituitary lesion were excluded in order to focus on clinically defined PAH. However, due to the retrospective design and lack of standardized headache phenotyping, complete differentiation between secondary tumor-related headache and primary headache disorders was not possible in all cases. Some overlap therefore cannot be entirely excluded. This limitation should be understood as inherent to retrospective real-world headache ascertainment rather than as a finding specific to one treatment group or tumor subtype.

With respect to treatment strategy, our data show that long-term headache outcomes are highly favorable after both upfront DA therapy and upfront TSS. Headache prevalence declined markedly over time in both groups, and persistent headache at last follow-up was rare overall. Given the retrospective design and non-random treatment allocation, these findings should not be interpreted as proof of therapeutic equivalence between medical and surgical management. Rather, they support a clinically more relevant conclusion: durable headache relief appears to be achievable with either approach when effective tumor control is obtained [47]. Both TSS and DA therapy ultimately reduce tumor burden and normalize prolactin levels, thereby addressing the underlying drivers of PAH. From a clinical perspective, these findings align with current management paradigms and with emerging evidence supporting a broader role for primary surgical treatment in selected prolactinoma patients. Although DAs remain first-line therapy for most prolactinomas, recent data indicate that upfront surgery may be an effective primary option in selected patients, particularly in those without cavernous sinus invasion [15–17, 48, 49]. In this context, our findings suggest that durable headache improvement can be achieved with both treatment strategies. Accordingly, in patients with clinically relevant headache, surgical treatment may represent a reasonable option within an individualized multidisciplinary decision-making process, particularly when patient preference, intolerance, or resistance to dopamine agonists are relevant considerations.

The observed overrepresentation of men among patients with PAH likely reflects later diagnosis and more invasive tumor characteristics rather than sex-specific susceptibility to headache [17, 48, 50, 51]. Male sex did not remain independently associated with PAH once invasiveness was taken into account. Accordingly, treatment decisions should be guided by tumor anatomy, endocrine behavior, and patient-specific factors rather than sex alone. Although earlier surgical consideration in men with prolactinomas has been proposed by some authors [52–54], our data do not support modifying established treatment algorithms solely for headache control.

### Limitations

This study has several limitations. First, its retrospective single-center design may introduce selection bias and limit generalizability. Second, headache status and outcome were derived from routine clinical documentation rather than from prospective standardized instruments, which may have resulted in information bias. Because headache was not assessed as a primary outcome in routine prolactinoma care, both the prevalence of prolactinoma-associated headache at diagnosis and the persistence of headache during follow-up may have been underestimated. This likely reflects real-world underrecognition rather than overreporting of headache burden and limited the assessment of headache phenotype, severity, and temporal course. In particular, detailed information on headache characteristics and on coexisting primary headache disorders was not systematically available, such that some degree of misclassification of headache etiology cannot be excluded. Furthermore, transient treatment-related worsening or exacerbation of headache was not assessed systematically. Third, treatment allocation was not randomized but was based on tumor characteristics, endocrine status, clinical presentation, and patient-related factors, introducing confounding by indication and limiting direct comparison between medical and surgical strategies. Although this reflects real-world clinical decision-making, residual confounding cannot be excluded. In addition, detailed Knosp grading was not consistently available across the entire cohort. Cavernous sinus invasion had originally been documented in the clinical database as a dichotomous variable, corresponding to the presence or absence of radiological cavernous sinus involvement. Because many of the original MRI studies were no longer available for systematic re-review over the long study period, reassessment of Knosp grades 1–4 across the full cohort was not feasible. Cavernous sinus involvement was therefore analyzed as a dichotomous variable, which may have limited our ability to assess whether more advanced invasion, such as higher Knosp grades, carries additional implications for headache severity, persistence, or treatment response.

Finally, quantitative longitudinal tumor volumetry was not available. We were therefore unable to directly assess parallel trajectories of tumor shrinkage and headache improvement over time or to provide a figure displaying tumor size throughout follow-up. This reflects an inherent limitation of the long-term real-world design of the study. In routine prolactinoma care, follow-up imaging is often performed less systematically once relevant tumor shrinkage has been documented and biochemical control has been achieved, with subsequent long-term follow-up relying predominantly on clinical assessment and prolactin measurements [10, 55, 56]. As a result, serial imaging data suitable for standardized volumetric analysis were not consistently available across the full cohort, particularly at later follow-up time points. In addition, because this cohort spans a long retrospective period, many original MRI studies were no longer fully available for systematic re-review, which also precluded retrospective reconstruction of longitudinal tumor volumetry. Although prolactin normalization represents a well-established surrogate of tumor control in prolactinomas, our findings should therefore be interpreted as demonstrating an association between successful prolactinoma treatment and headache improvement rather than proving a direct causal relationship between radiological tumor shrinkage and headache remission.

## Conclusions

Headache is a common and clinically relevant presenting symptom in prolactinoma patients and is most strongly associated with tumor invasiveness. Effective prolactinoma treatment is associated with substantial and durable headache relief under both upfront medical and upfront surgical management. These findings support careful patient counseling and suggest that individualized prolactinoma management is likely to result in sustained headache improvement in most patients.

## Data Availability

The authors agree to share data on request.
